# Controlled Synthesis of Polyphosphazenes with Chain-Capping Agents

**DOI:** 10.3390/molecules26020322

**Published:** 2021-01-10

**Authors:** Robert A. Montague, Krzysztof Matyjaszewski

**Affiliations:** Department of Chemistry, Carnegie Mellon University, 4400 Fifth Avenue, Pittsburgh, PA 15213, USA; rmontague.ky@roadrunner.com

**Keywords:** *N*-alkyl phosphoranimines, polyphosphazenes, phosphoramidate esters, fluoride, trifluoroethoxide, *N*-methylimidazole initiators, anionic, chain-capping agents

## Abstract

*N*-alkyl phosphoranimines were synthesized via the Staudinger reaction of four different alkyl azides with tris(2,2,2-trifluoroethyl) phosphite. *N*-adamantyl, *N*-benzyl, *N*-*t*-butyl, and *N*-trityl phosphoranimines were thoroughly characterized and evaluated as chain-capping compounds in the anionic polymerization of *P*-tris(2,2,2-trifluoroethoxy)-*N*-trimethylsilyl phosphoranimine monomer. All four compounds reacted with the active chain ends in a bulk polymerization, and the alkyl end groups were identified by ^1^H-NMR spectroscopy. These compounds effectively controlled the molecular weight of the resulting polyphosphazenes. The chain transfer constants for the monomer and *N*-benzyl phosphoranimine were determined using Mayo equation.

## 1. Introduction

The most important examples of inorganic polymers [1,2,3] are the polysiloxanes—(R_2_Si-O)_n_ [4,5,6,7,8], polysilanes—(R_2_Si)_n_ [9,10,11], and the polyphosphazenes—(R_2_P=N)_n−_ [12,13,14]. They have been the subject of significant research due to desirable properties, such as biocompatibility, high thermal resistance, oxidative stability, UV resistance, interesting photoelectronic behavior, and high flexibility that are often difficult or impossible to achieve in carbon-based organic polymers. Much of the current research on polyphosphazenes is focused on applications in the life sciences as drug delivery vehicles and other biological applications [15,16,17,18].

The field of polyphosphazene synthesis has expanded significantly with the advent of more efficient and faster synthetic routes such as the ambient temperature preparation of poly(dichlorophosphazene) by the PCl_5_-initiated polymerization of *N*-trimethylsilyl-*P*-trichlorophosphoranimine, [13,19] the polymerization of *N*-trimethylsilyl-*P*-tris(2,2,2-trifluoroethoxy)phosphoranimine by antimony pentachloride initiator, [20] and poly(organophosphazenes) from partially halogenated phosphoranimines [21]. These reactions are examples of cationic polymerization of phosphoranimines. The cationic route has also led to polyphosphazenes with functional and terminal groups at the chain ends [22,23,24,25,26,27] as well as many other derivative copolymers with more complex architecture [28,29,30,31,32].

**Scheme 1:**
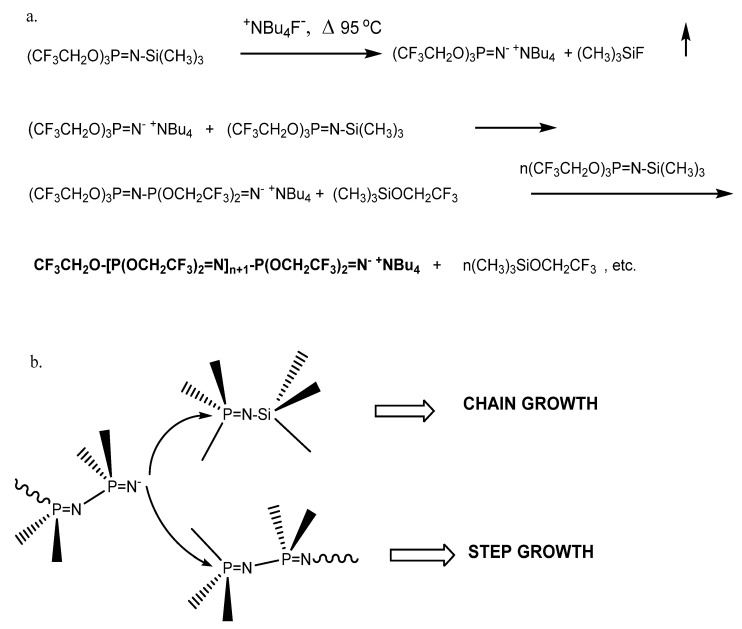
(**a**) Anionic chain growth condensation polymerization. (**b**) Chain growth vs. step growth macrocondensation.

The first catalyzed/initiated polymerization of a phosphoranimine monomer by fluoride ion was previously reported (Scheme 1a) and then extensively investigated [33]. The successful conversion of these monomers to polyphosphazenes initiated/catalyzed by fluoride anion, trifluoroethoxide anion, and *N*-methylimidazole was described [34,35,36]. Polyphosphazene random and block copolymers with mixed alkoxy/alkoxyether substituents were subsequently prepared by the fluoride/anionic method and characterized [37]. Other examples include polyphosphazenes with alkyl or aryl substituents [38], polyphosphazenes with electronegative nitropropoxy groups bound to the phosphorus atom of the *N*-silylated phosphoranimine [39], polymerization of *N*-silyl-*P*-diethyl phosphoranimine with fluoride and phenoxide initiators [40], and *P*-tris(trifluoroethoxy)-*N*-trimethylsilyl phosphoranimine polymerized with water in the presence of *N*-methylimidazole initiator in diglyme solution polymerization [41].

The use of chain capping agents is a technique to prepare well-defined polyphosphazenes produced by anionic polymerization of phosphoranimines, since active chain segments are subject to late-stage condensation reactions that can increase molecular weight with diminished control of chain length. In Scheme 1b, active anionic chain ends of the forming polymer can react with a monomer molecule via chain growth condensation (with elimination of trimethylsilyl trifluoroethoxide), or it may react with a partially polymerized chain segment to advance molecular weight via a macrocondensation. The preparation of four *N*-alkyl phosphoranimine compounds and their efficiency as chain end-capping agents in polyphosphazene synthesis was briefly mentioned before [42].

In this paper, we report more detailed experimental data obtained using these compounds, and present evidence of their incorporation as unique chain end groups during the formation of polyphosphazenes by the anionic route.

## 2. Results and Discussion

### 2.1. Synthesis and Characterization of N-Alkyl Phosphoranimines

**Scheme 2:**
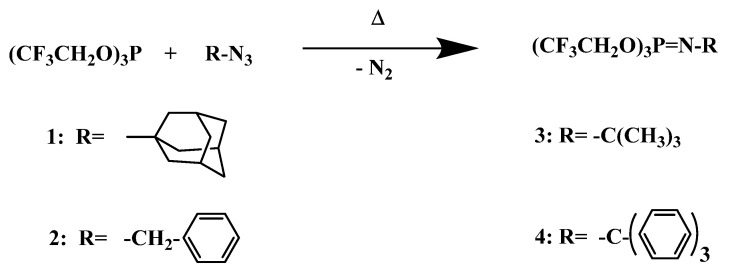
Staudinger synthesis of *P*-tris(trifluoroethoxy)-*N*-alkylphosphoranimines.

The synthetic route to the four *N*-alkyl phosphoranimines prepared in this study was via the Staudinger reaction [43] is shown in Scheme 2, in which tris(2,2,2-trifluoroethyl) phosphite reacted with an alkyl azide to form the *P*-tris(2,2,2-trifluoroethoxy)-*N*-alkyl phosphoranimine with the evolution of nitrogen gas.

The *N*-alkyl compounds formed consisted of the *N*-adamantyl, *N*-benzyl, *N*-*t*-butyl, and *N*-trityl phosphoranimines. Yields, boiling or melting points, densities, and refractive indices are shown in Table 1.

The new compounds were characterized by NMR, fast atom bombardment, and GC-mass spectrometry, with the resulting data presented in Table 2. The data obtained from the NMR spectra are consistent with the proposed structures of the four compounds, as are the mass spectrometry measurements for molecular weight of the parent ions.

Elemental analyses of these compounds for found and theoretical percentages are displayed in Table 3. The determined percentages of elements from the analyses are in good agreement with the assigned structures.

**Figure 1:**
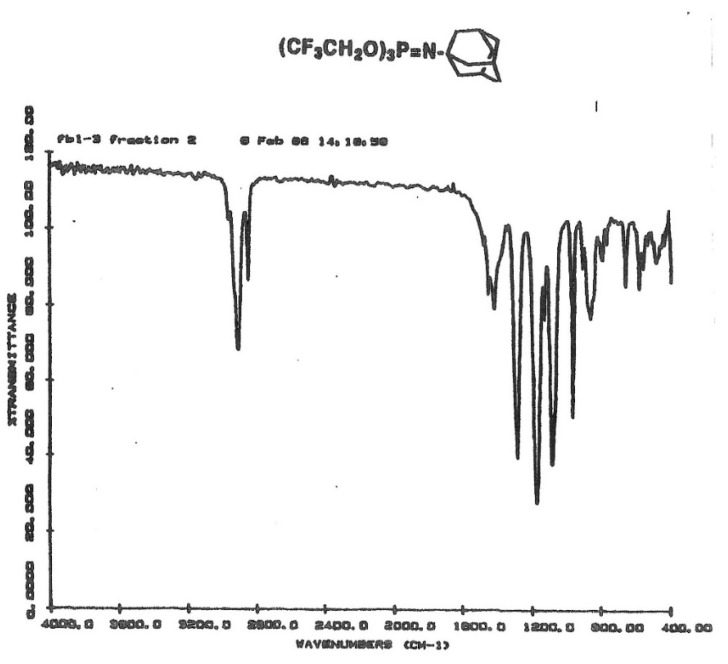
FT-IR spectrum of *P*-tris(trifluoroethoxy)-*N*-adamantyl phosphoranimine.

FTIR spectra further confirmed the structures with the appearance of the broad absorption bands of the P=N moieties between 1270–1310 cm^−1^, and the characteristic absorptions of aliphatic or aromatic protons as expected for the particular structure. The data collected are shown in Table 4, and an example IR spectrum of the *N-*adamantyl phosphoranimine is shown in Figure 1.

### 2.2. Reactivity of N-Alkyl Phosphoranimines

The phosphoranimines are air-sensitive and hydrolyze easily to the corresponding phosphoramidite esters, (CF_3_CH_2_O)_2_P(=O)-NHR, as confirmed by mass spectrometry and NMR. For example, the GC-mass spectrum of the *N*-adamantyl phosphoranimine in (wet) diethyl ether showed two major peaks with fragments that are consistent for the phosphoranimine (molecular ion 477), and the corresponding *N*-adamantyl phosphoramidate ester (molecular ion 395). A third peak in trace amount is in good agreement with the 1-azidoadamantane starting reagent, and all three species contain the adamantyl fragment (135).

The proton-coupled ^31^P-NMR spectrum of the GC-MS sample showed the phosphoranimine signal at −27.4 ppm, and the new species at +7.0 ppm. The new signal is a sextet with a coupling constant of 7.3 Hz, as expected for ^3^*J*_POCH_ coupling.

The ^1^H-NMR spectrum of this sample showed the ethyl ether proton signals at 1.18 ppm (t) and 3.45 ppm (q); two distinct sets of the different adamantyl protons from 1.58 to 2.10 ppm; amidate proton at 2.80 ppm (d); and trifluoroethoxy protons at 4.27 ppm (p). The amidate doublet has a coupling constant of ^2^*J*_PNH_ = 9 Hz. This data supports the structure assignment for the hydrolysate.

**Scheme 3:**
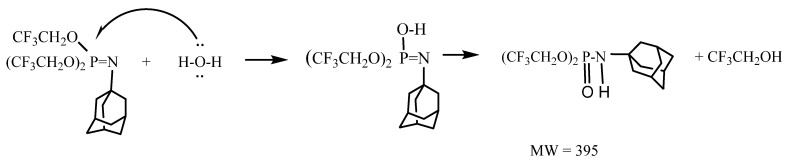
Reaction of *P*-tris(trifluoroethoxy)-*N*-adamantylphosphoranimine with H_2_O.

The phosphoramidate ester can arise from nucleophilic attack by water (or OH^−^) on the electrophilic phosphorus atom of the phosphoranimine, as shown in Scheme 3.

Similar behavior was reported in the study of an *N*-silylated phosphoranimine [44]. This could be a general reaction of phosphoranimines bearing trifluoroethoxy (or other leaving) groups bound to the phosphorus center.

A sample of the *N*-benzyl phosphoranimine was exposed to air and was converted to a colorless crystalline solid with a δp = +8.3 ppm, which is consistent with the corresponding *N*-benzyl phosphoramidate ester, (CF_3_CH_2_O)_2_P = (O)-NHCH_2_Ph [45].

In addition to the phosphoramidate ester major product, a trace signal at −152.5 ppm was also observed. This high field resonance arises from the hexacoordinate species (CF_3_CH_2_O)_6_P^−^, in which the phosphorus center carries a formal negative charge. This compound was previously prepared and its ^31^P-NMR chemical shift was reported as −154.6 ppm [46]. Electronegative groups stabilize the phosphorus hexacoordinated state [47].

This species has also been observed as a trace component of polymerizing mixtures of the *P*- tris(2,2,2-trifluoroethoxy)-*N*-trimethylsilyl phosphoranimine monomer, and in unreacted fractions of tris(2,2,2-trifluoroethyl) phosphite that were distilled from reaction mixtures. It is probably formed by a thermal rearrangement of phosphorus compounds bearing trifluoroethoxy ligands.

A pentacoordinated phosphorus compound was observed in the ^31^P-NMR spectrum of a sample of the clear colorless *N*-benzyl phosphoranimine that had crystallized after several weeks in a desiccator. Its chemical shift of −72 ppm is typical of five-coordinate phosphorus compounds as previously reported [45], and sufficiently electronegative ligands tend to stabilize such species [45]. The penta-(2,2,2-trifluoroethoxy) phosphorane, P(OCH_2_CF_3_)_5_, was previously prepared and its ^31^P-NMR chemical shift reported as −76.6 ppm [46]. Another example of a penta-coordinated phosphorus compound with an *N*-benzyl ligand is (CF_3_)_3_ (F)P[N(CH_3_) (CH_2_Ph)] with a δp = −68.8 ppm [48].

**Scheme 4:**
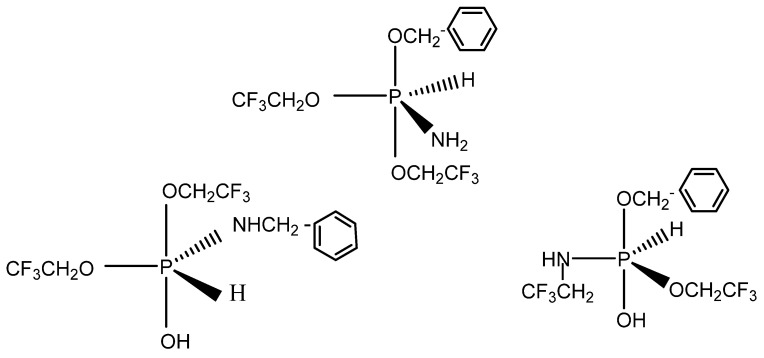
Pentacoordinated phosphorus structures.

The mass spectrum of the crystallized sample of the *N*-benzyl phosphoranimine showed a high mass peak of 351. The pentacoordinate phosphorus compounds shown in Scheme 4 are potential isomers with MW = 353 and are reasonable structural assignments for the species observed. They may exist in equilibrium with their phosphonium salts of the general formula, R_4_P^+^R^−^ [45].

### 2.3. Polymerization Studies

#### Bulk Polymerization with Addition of *N*-alkyl Phosphoranimines

Experiments were conducted in which small samples of these compounds were added to polymerizing mixtures of the tris(2,2,2-trifluoroethoxy) phosphoranimine monomer and corresponding polyphosphazene. The initial experiment probed the reactivity of the *N*-alkyl phosphoranimine in bulk polymerization. *P*-tris(2,2,2-trifluoroethoxy)-*N*-trimethylsilyl phosphoranimine monomer, 1.5 mmol (CF_3_CH_2_O)_3_P = N-Si(CH_3_)_3_, was treated with 1 mol% tetrabutylammonium fluoride (TBAF) in a dry NMR tube and heated at 150 °C for 15 min. Then, 0.6 mmol of *N*-benzyl phosphoranimine was injected by syringe, mixed, and heated for another 15 min.

The reaction was cooled to room temperature, and ^31^P-NMR spectra confirmed polymer formation, along with a few percent oligomers. There were two very small doublets (+8.5, −2.5 ppm, *J*_PNP_ = 73 Hz) in the spectrum of the reaction mixture (less than 2%) which indicate formation of a phosphazene dimer as a minor side product, probably formed by a coupling reaction of monomer and the *N*-benzyl compound. A small signal at −72 ppm indicated the presence of ca. 2% of the pentacoordinate phosphorane discussed above.

The sample was then heated for an additional 15 min and final spectra recorded. A control reaction without *N*-benzyl phosphoranimine was run concurrently. The polymer samples were dissolved in diglyme and precipitated from excess cold chloroform, then re-dissolved in diglyme and precipitated again from 90/10 CHCl_3/_MeOH with thorough washing of the white polymer solids.

**Figure 2:**
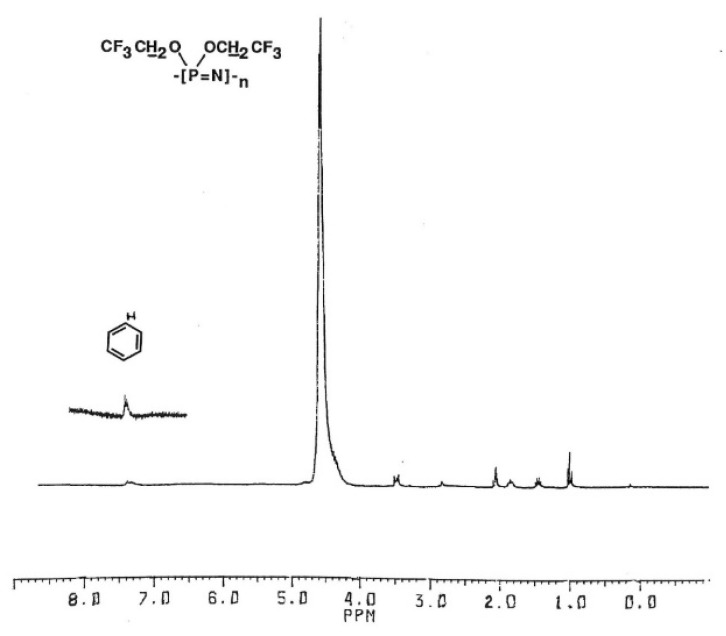
^1^H-NMR spectrum of *N*-benzyl-capped poly(bis(trifluoroethoxy)phosphazene).

The ^1^H-NMR spectra of the isolated polymer in d_6_-acetone solution showed the polymer signal at 4.55 ppm, *n*-butyl protons from the TBAF initiator, and new signals at 7.35 ppm indicating the aromatic protons of the benzyl group (Figure 2).

The ^31^P-NMR spectrum of this sample showed the polymer signal at −7.05 ppm, but no signals for the *N*-benzyl phosphoranimine, dimer, or phosphorane. The aromatic proton signals were thus ascribed to reaction of the *N*-benzyl compound with the polymer chain. Integration of the polymer and benzyl proton signal ratio correlated with incorporation of one *N*-benzyl phosphoranimine residue per chain, indicative of a functionalized chain-end. Similar results were obtained using the other *N*-alkyl phosphoranimines. After heating 6 mmol of monomer and 1 mol% TBAF initiator for 15 min at 150 °C, 3 mmol of the *N*-alkyl compound (neat or in solution) was added by syringe and heating continued for a total time of 1 h.

**Figure 3:**
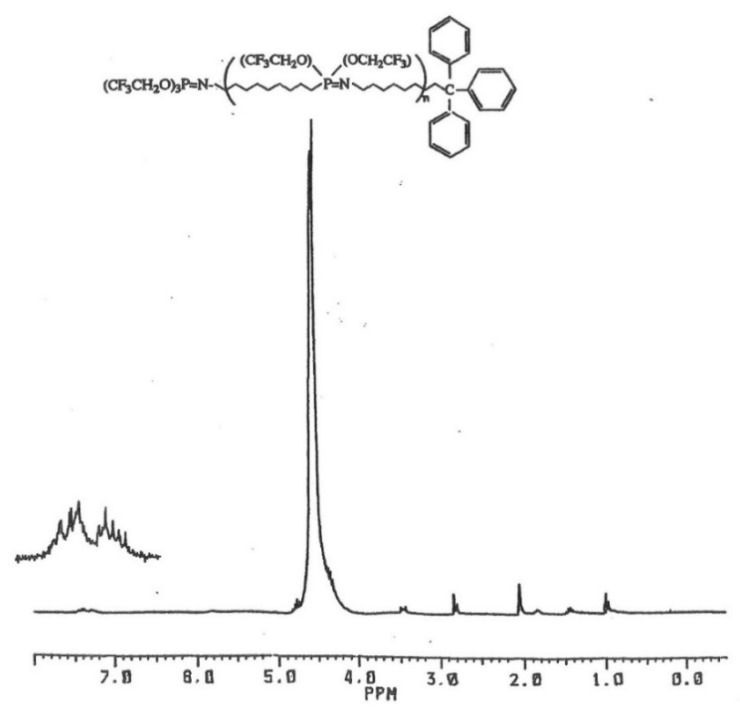
^1^H-NMR spectrum of *N*-trityl-capped poly(bis(trifluoroethoxy)phosphazene).

The polymer samples were precipitated twice as described before, and the ^1^H-NMR spectra showed signals for polymer, the *n*-butyl groups of the TBAF, and the corresponding alkyl group. The presence of the trityl-capped chains is particularly distinctive by the multiple aromatic proton signals of the formed polymer product, as seen in Figure 3. The proton signals for the adamantyl and *t*-butyl chain-end groups are partially obscured by the TBAF initiator residue, but they can be seen and identified. The ^31^P-NMR spectra of the experimental polymer samples showed no signals for unreacted *N*-alkyl phosphoranimines. The control reaction of monomer and TBAF showed proton signals for polymer and *n*-butyl groups, and GPC confirmed higher molecular weight. The yield of the polymer was significantly higher, indicating a more complete monomer conversion.

The molecular weights of the polymer samples obtained show the effect of the addition of the *N*-alkyl compounds. As seen in Table 5, the molecular weight and yield of polymer were significantly reduced by the addition of the *N*-alkyl compound compared to the control.

In related experiments, bulk polymerizations with monomer-equivalent additions of *N*-benzyl phosphoranimine (NBP) at specific intervals during 150 °C polymerization using 1 mol% TBAF initiator showed that the polymer molecular weight was terminated upon addition compared to controls (*) with no *N*-benzyl compound (Table 6). Neither conversion nor molecular weight increased after addition of the NBP.

The effect of various amounts of *N*-benzyl phosphoranimine was examined by preparing samples containing monomer, 1 mol% TBAF initiator, and *N*-benzyl compound in the ratios shown in Table 7. After heating at 150 °C for 45 min, the molecular weights of the polymers obtained were measured by GPC and conversion determined by ^31^P-NMR spectra integration. These data show that at least 2% *N*-benzyl phosphoranimine was needed to effectively limit polymer molecular weight and conversion of monomer, with 20% inhibiting polymerization.

The polymer molecular weight, dispersity, and yield are decreased by the *N*-alkyl phosphoranimine addition. However, the data also show that perfect stoichiometric agreement between the amount of the capping agent and degree of polymerization (DP) was not always observed. For example, the 20:1 and 50:1 samples have DPs of about 20 and 50, respectively, but the other ratios exhibit some variability. The lower than expected DP of the 100:1 sample may arise from other transfer reactions in the system.

**Figure 4:**
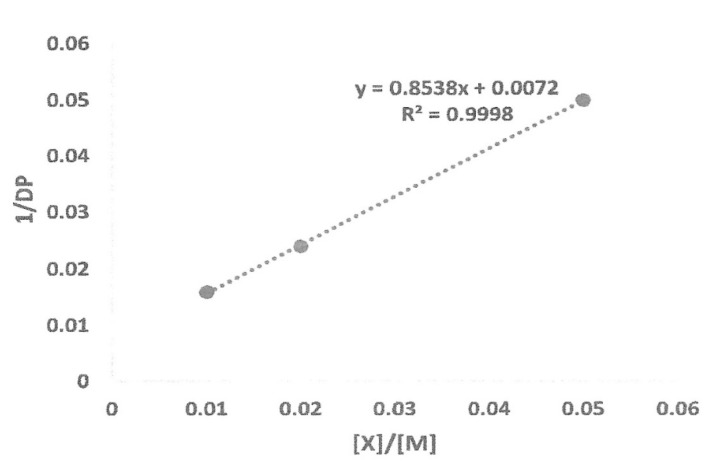
Mayo plot of 1/DP as function of [X]/[M] ratio.

Using the data from Table 7, a graph was constructed, as shown in Figure 4. The following Mayo equation was used to determine the chain transfer to the capping agent constant, C_tr,X_, and the constant for transfer to monomer, C_tr,M_:

1/DP_n_ = C_tr,M_ + C_tr,X_ [X]/[M]

From the graph, the slope of the line is the chain transfer constant to capping agent and the y-intercept is the chain transfer to monomer constant. From the equation, the values for C_tr,X_ and C_tr,M_ are 8.54 × 10^−1^ and 7.2 × 10^−3^_,_ respectively. There was a very small signal from trimethylsilyl protons in the ^1^H-NMR spectrum of precipitated polymer at 0.12 ppm (Figure 2), which indicated that some chains could be terminated by trimethylsilyl groups, albeit in trace amount.

A final experiment was conducted in which a 1:1.7 mol mixture of monomer and *N*-adamantyl phosphoranimine with 1% (mol) TBAF was heated for several hours at 150 °C, well beyond the normal time required for complete conversion of monomer to polymer in the presence of the fluoride. Analysis of the reaction mixture by ^31^P-NMR showed that only 13% conversion of the monomer occurred, producing polymer and oligomers, with 87% of the spectrum signals arising from unreacted monomer and the *N*-adamantyl compound. Under these forcing conditions, the polymerization was retarded to a major extent, but not completely inhibited by the *N*-adamantyl phosphoranimine. The amount of polymer in the reaction mixture was less than 7% and could not be isolated for inspection of end groups.

Significantly, no phosphorus doublets were observed in the ^31^P-NMR spectrum, thus, the monomer and *N*-adamantyl compound did not react to form a phosphazene dimer. This result and that of the end-capping experiments indicate that the *N*-adamantyl phosphoranimine reacts preferentially with active chain ends.

**Scheme 5:**
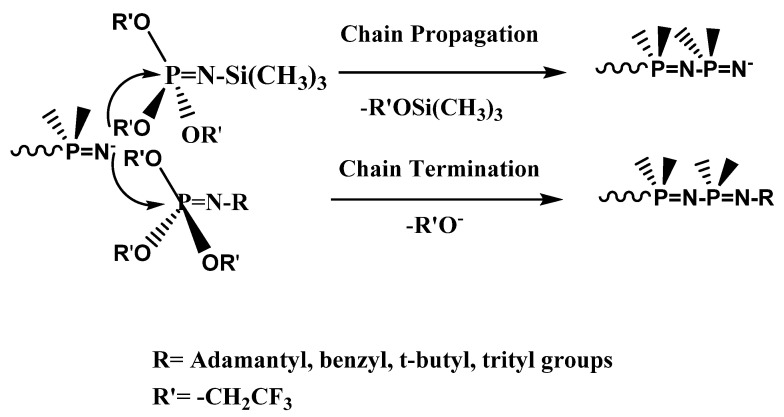
Chain propagation vs. chain termination.

The active species, particularly in the early chain-growth stage of the polymerization, should be an anionic polymer chain-end [49]. The phosphorus atom of the *N*-alkyl phosphoranimine has similar reactivity as in the *N*-silylated monomer (C_tr,X_ ~1) but forms an unreactive group. The stable alkyl group of the new phosphoranimine leads to termination of the chain, whereas the labile silyl group of the monomer results in continued propagation of the chain, as shown in Scheme 5.

Chain termination with these compounds is a degradative transfer reaction in which an active chain is terminated and a new active species, trifluoroethoxide anion, is produced. The number of active species in the system is unchanged, and the anion can initiate a new chain, as reported in an earlier study [49,50].

## 3. Conclusions

Four *N*-alkyl phosphoranimines were synthesized and found to be effective chain capping agents for polyphosphazenes prepared by anionic initiation of a phosphoranimine monomer. The NMR and GPC data support their incorporation into the chain as end groups. The use of chain-capping/terminating compounds in the anion-initiated polymerization of a phosphoranimine monomer has been demonstrated and may provide a useful technique for further functionalization of polyphosphazenes. For example, it may be possible to add alkenyl groups to short phosphazene chain ends as a route to prepare inorganic/organic graft copolymers, as well as to prepare block copolymers with controlled molecular weight.

## 4. Materials and Methods

### 4.1. Materials

Tris(2,2,2 trifluoroethyl) phosphite and trimethylsilyl azide were obtained from Aldrich Chemical Co., Milwaukee, WI, USA and purified by distillation at reduced pressure from 4 Angstrom molecular sieves to molecular sieves. Tetra-*n*-butyl ammonium fluoride TBAF in THF, 1.0M and TBAF-silica gel were obtained from Aldrich and were used as received. Benzyl azide was obtained from Johnson-Mathey Co., London, England and purified by recrystallization from dry benzene-hexane solution. 1-Azidoadamantane and sodium azide from Aldrich were used as received. Trityl bromide and *t*-butyl alcohol (Fisher ScientificFisher Scientific Co., LLC Hampton, NH, USA), and BF_3_.ET_2_O from Aldrich were used as received.

### 4.2. Monomer Synthesis

A clean 100 mL three-neck round bottom flask with magnetic stir-bar, water jacketed condenser, glass or Teflon stopcock, and Claisen tube with 250 mL pressure-equalizing addition funnel was oven-dried at 150 °C overnight, assembled hot with natural rubber septa and gas inlet and outlet, and cooled under a dry nitrogen purge. All ground-glass joints were sealed with a light application of stopcock grease and wrapped with Teflon tape. The flask was covered with aluminum foil to prevent photolysis of the azide. In accordance with a literature procedure [51], distilled tris(2,2,2-trifluoroethyl) phosphite (0.4 mol) was combined with equimolar distilled trimethylsilyl azide and refluxed for 24 h at 120 °C, followed by two successive additions of equimolar azide under agitation at 24 h intervals, for a final mole ratio of 1:3 phosphite:azide. During each azide addition, the reaction flask was cooled to 0 °C, then the temperature was slowly raised to 120 °C. At the end of the 72-h period, an orange/amber liquid was observed in the flask. Upon distillation at reduced pressure, a first fraction of unreacted clear, colorless azide was obtained at 40–50 °C/85 mm, and a second fraction of clear, colorless liquid monomer distilled at B.P. 50–60 °C/0.5 mm. Yield of *P*-tris (2,2,2 trifluoroethoxy)-*N*-trimethylsilyl phosphoranimine monomer: 95% based on the phosphite.

### 4.3. Synthesis of N-Alkyl Phosphoranimines

The adamantyl azide (21 mmol) and an equimolar amount of the tris(2,2,2-trifluoroethyl) phosphite were combined in a three-neck round bottom 100 mL flask with condenser over an electrically heated and thermostated oil bath. The oil temperature was increased gradually from 50 °C to 140 °C over a 1.75 h period, in order to moderate the pressure increase in the system as the nitrogen outgassed. The *N*-adamantyl phosphoranimine distilled under reduced pressure (93 °C/2.5 torr) as a clear, colorless liquid which crystallized as colorless needles with a melting point near 0 °C as shown in Table 1.

In a similar fashion, 136 mmol benzyl azide and equimolar amount of phosphite were combined with stirring at 40–80 °C for 22 h. ***CAUTION: very vigorous reaction with rapid outgassing and pressure build-up in apparatus*!** *N*-benzyl phosphoranimine distilled as a clear colorless liquid at 95 °C/2.5 torr. The *t*-butyl azide was prepared by the reaction of 100 mmol *t*-butyl alcohol and 120 mmol trimethylsilylazide in the presence of 120 mmol BF_3_.Et_2_O, following a published procedure [52]. Due to difficulty in isolating the azide by distillation, 100 mmol phosphite was added to this reaction at room temperature (RT) for the Staudinger coupling in situ, at 90 °C for 18 h. *N*-*t*-butyl phosphoranimine distilled from the reaction as a clear, colorless liquid. Trityl azide was prepared by the reaction of sodium azide suspended in MeCN with trityl bromide in benzene at RT over several days, in a manner similar to published methods [53,54]. The crystallized azide (13.5 mmol) was combined with an equimolar amount of phosphite at 60–140 °C over a 2-h period. *N*-trityl phosphoranimine crystallized as an amber, fibrous solid in high purity.

### 4.4. Bulk Polymerization of Monomer and N-Alkyl Phosphoranimines

The bulk polymerizations of the monomer phosphoranimine with and without the *N*-alkyl compounds were conducted in NMR tubes as indicated in the Results and Discussion section. Monomer in the specified amount was charged via syringe and the specified amount of TBAF initiator solution was added by microliter syringe. Heat was supplied by a mineral oil bath over an RCT Tekmar hotplate unit for the specified time period. The *N*-alkyl phosphoranimine was added neat if liquid, or as a solution by syringe as indicated. After the reaction was cooled to RT, the work-up entailed dissolving the solid polymer mass in 2–5 mL THF, and adding the solution to excess cold chloroform to precipitate the polymer. The polymer mass was re-dissolved in a 90/10 blend of chloroform and methanol and re-precipitated. The re-precipitated polymer was allowed to stand under the mother liquor at −20 °C overnight to maximize precipitation, and was collected on a clean, tared glass frit with thorough washing to remove any unreacted materials. The collected white polymer solid was dried in a vacuum desiccator overnight before weighing. Yield was calculated on the basis of monomer weight minus the condensate by-product as the theoretical yield.

### 4.5. Characterization of N-Alkyl Phosphoranimines and Polymers

NMR spectra were recorded on an IBM NR/300 MHz FT NMR spectrometer. Trimethyl phosphite in C_6_D_6_ was used as an external standard for ^31^P-NMR spectra, with a δ P of 141.0 ppm (85% phosphoric acid H_3_PO_4_ = 0 ppm). The ^31^P-NMR and ^1^H-NMR spectra were obtained in CDCl_3_ solution or in d^6^ acetone as internal standard (^1^H-NMR = 7.24 ppm). Abbreviations for NMR signals: s = singlet; m = multiplet, d = doublet, t = triplet, q = quartet, p = pentet, br. = broad. Integration symbols, such as “6H”, signify six protons of a particular type as shown in the table.

Mass spectra were obtained using a Hewlett Packard 5890 gas chromatograph with a silica column and equipped with a 5970 series mass spectrometer. Run time was 40 min with 2 min solvent delay; T (initial) = 100 °C for 10 min, heating rate of 10 °C/minute, and T (final) = 250 °C for 5 min. FTIR spectra were obtained on a Nicolet 5DXB FT-IR spectrometer. Samples were analyzed in KBr pellets, or thin translucent films between sodium chloride plates as appropriate, using polystyrene film standard. Melting points were measured with a digital melting point apparatus from Electrothermal Eng. Ltd. at a heating rate of 1 °C/minute. Refractive indices were measured on the Bausch and Lomb Abbe’−3L refractometer at 20 °C. Elemental analyses were provided by Midwest Microlab of Indianapolis, IN. Gel Permeation Chromatography (GPC) was performed on polymer samples by first dissolving the solid polymer (0.2–0.5 g) in 1 mL THF-HPLC grade, and filtering through 0.5 micron Teflon filter, 20 microliters of this solution was injected into the carrier solvent stream (THF with 0.1% tetra-*n*-butyl ammonium bromide, a literature procedure for polyphosphazenes [55]) at a flow rate of 1.0 mL/minute. Ultrastyragel columns (10,000; 1000; 100 Angstroms) and a Waters 410 differential refractometer were used at 35 °C internal temperature. Data acquisition and calculations were performed with a Nelson 900 analytical interface and Samsung 286 personal computer. Calibration was based on polystyrene standards of low to high molecular weights.

## Figures and Tables

**Table 1:** Physical data for *N*-alkyl phosphoranimines.

Compound	% Yield	B.P.(M.P.)	Density	Refractive Index, n_D_^20^
**1** (Adamantyl)	46.3	93 °C/2.5 torr, (0–2 °C)	1.606 g/mL	1.4076
**2** (Benzyl)	98.9	95 °C/2.5 torr	1.664 g/mL	1.4022
**3** (*t*-Butyl)	34.4	93 °C/52 torr	1.568 g/mL	1.3445
**4** (Trityl)	56.5	(80–85 °C)		

**Table 2:** NMR and mass spectrometry data for *N*-alkyl phosphoranimines.

Compound.	^31^P-NMR, ppm	^1^H-NMR ppm	Mass Spectrometry	Theor. MW
**1** (Adamantyl)	−27.3 (m)	4.28 (p): 6H ^3^*J*_(POCH)_ = 7.6 Hz 1.73(d): 6H 1.65(br. S.):6H 2.05(br. s.):3H	477 **	477
**2** (Benzyl)	−13.19 (m)	4.20 (p): 6H ^3^*J*_(POCH)_ = 7.2 Hz 4.32 (d): 2H^3^*J*_(PNCH)_ = 22 Hz 7.30 (m): 5H	433 ^	433
**3** (*t*-Butyl)	−28.95 (m)	4.28 (p): 6H^3^*J*_(POCH)_ = 8.2 Hz 1.23 (s): 9H	399 *	399
**4** (Trityl)	−29.01 (p)	4.05 (p): 6H^3^*J*_(POCH)_ = 7.8 Hz 7.31(m): 15H	585 **	585

* Molecular ion; ^ Partially decomposes on silica GC column; ** Molecular ion, FAB mass spec.

**Table 3:** Elemental analyses of *N*-alkyl phosphoranimines.

Compound	% C	% F	% H	% N	%O	%P	Theory/Found
**1** (Adamantyl)	40.28	35.83	4.45	2.94	10.06	6.47	Theory
	40.58	35.49	4.54	3.22	-	6.33	Found
**2** (Benzyl)	36.06	39.47	3.03	3.24	11.8	7.15	Theory
	35.73	38.12	2.98	3.16	-	6.77	Found
**3** (*t*-Butyl)	30.10	42.83	3.80	3.51	12.03	7.76	Theory
	29.36	41.88	3.65	3.27	-	7.75	Found
**4** (Trityl)	51.32	29.22	3.63	2.39	8.21	5.29	Theory
	48.14	23.89	3.60	2.25	-	*	Found

Notes: Fluorine interferes with oxygen determination. * Sample size insufficient for P-analysis.

**Table 4:** FTIR band assignments of *N*-alkyl phosphoranimines.

Compound	Absorbance Wavenumbers cm^−1^	Functional Group Assignment
**1** (Adamantyl)	2870–2950	CH aliph.
	1435	P-O-C
	1270	P=N
	1180, 960	P-O
	1090	C-O
	660	CF_3_
**2** (Benzyl)	3020–3120	CH arom.
	1305	P=N
	1150, 970	P-O
	1090	C-O
	700–750	CH arom.
	670	CF_3_
**3** (*t*-Butyl)	2970	CH aliph.
	1420	P-O-C
	1310	P=N
	1170, 970	P-O
	1085	C-O
	675	CF_3_
**4** (Trityl)	3000–3110	CH arom.
	1420–1440	P-O-C
	1300	P=N
	1180	P-O
	1090	C-O
	720–750	CH arom.
	660–670	CF_3_

**Table 5:** Molecular weights, dispersity, yields of *N*-capped polyphosphazenes (bulk polymerization at 150 °C 1% TBAF, 3 mmol *N*-alkyl phosphoranimine addition, heated for 1 h).

Polymer End Group	M_n_	M_w_/M_n_	% Grav. Yield
Adamantyl	21,960	1.40	22
Benzyl	19,867	1.34	25
*t*-Butyl	21,297	1.49	34
Trityl	17,362	1.51	33
Control (no *N*-alkyl)	29,012	1.70	71

**Table 6:** Effect of addition time of *P*-Tris(2,2,2-trifluoroethoxy)-*N*-trimethylsilyl phosphoranimine on conversion and molecular weight (bulk at 150 °C, 1% TBAF).

NBP Sample	Minutes before Add.	Time, Min.	M_n_	M_w_/M_n_	% Conversion
**1**	2	20	7123	1.08	9
2 *	--	2	9757	1.29	35
**3**	5	20	11,472	1.45	29
4 *	--	5	15,646	1.40	54
5 *	--	20	18,188	1.72	81

* controls, no *N*-benzyl phosphoranimine.

**Table 7:** Effect of various monomer: *N*-benzyl phosphoranimine ratios on conversion and molecular weight.

Monomer: *N*-Benzyl mol. Ratio	M_n_	M_w_/M_n_	% Conversion
5:1	Polymer not detected	--	--
20:1	4849	1.02	7
50:1	10,183	1.50	52
100:1	14,952	1.73	86
Control	15,195	1.84	86

## Data Availability

The data presented in this study are available in this article.

## Sample Availability

Samples of the compounds are not available from the authors.
